# Preventive Effects of Fluoro-Substituted Benzothiadiazole Derivatives and Chitosan Oligosaccharide against the Rice Seedling Blight Induced by *Fusarium oxysporum*

**DOI:** 10.3390/plants8120538

**Published:** 2019-11-24

**Authors:** Bo Ma, Junhe Wang, Chuanzeng Liu, Jifang Hu, Kefei Tan, Fuyang Zhao, Ming Yuan, Junhua Zhang, Zhijia Gai

**Affiliations:** 1 College of Agriculture, Northeast Agricultural University, Harbin 150030, China; mabo8210@163.com; 2 Qiqihar Branch of Heilongjiang Academy of Agricultural Sciences, Qiqihar 161006, China; wangjunhe63@163.com (J.W.); cjf69@163.com (C.L.); hujifang7@163.com (J.H.); tkfhlj@163.com (K.T.); zfyhhh@126.com (F.Z.); 55677909@163.com (M.Y.); 3 Jiamusi Branch of Heilongjiang Academy of Agricultural Sciences, Jiamusi 154007, China; gaizhijia@163.com

**Keywords:** rice, *Fusarium oxysporum*, FBT, COS, momilactone

## Abstract

Rice seedling blight, caused by *Fusarium oxysporum*, significantly affects global rice production levels. Fluoro-substituted benzothiadiazole derivatives (FBT) and chitosan oligosaccharide (COS) are elicitors that can enhance plant resistance to pathogen infection. However, there is a lack of information regarding FBT and COS used as elicitors in rice seedlings blight. Therefore, the aim of this study was to evaluate the effect of FBT and COS treatments on rice seedling blight and elucidate the molecular mechanisms of the two elicitors for inducing resistance using proteomic technique. Results indicated that FBT and COS significantly reduced the disease incidence and index, and relived the root growth inhibition caused by *F. oxysporum* (*p* < 0.05). Biochemical analyses demonstrated that these two elicitors effectively enhanced activities of defense enzymes. Moreover, the proteomic results of rice root tissues disclosed more differentially expressed proteins in diterpenoid biosynthesis pathway that were particularly stimulated by two elicitors compared to the other pathways studied, resulting in the accumulation of antimicrobial substance, momilactone. Findings of this study could provide sound theoretical basis for further applications of FBT and COS used as rice elicitors against seedling blight.

## 1. Introduction

Rice (*Oryza sativa* L.) is an important global cereal crops that provides a stable food supply for more than 5 billion people worldwide. Most Asian diets include rice as a main dish [1]. Although global rice demand is on the rise, its productivity is constrained by disease outbreaks. One major fungal disease that occurs during the nursery and field planting stages is the rice seedling blight. Grain yield losses due to seedling blight infection ranges from 8 to 50% depending on severity of the disease, stage of the crop at which it was infected by the fungus, and overall environmental conditions [2,3,4]. Although several species belonging to *Fusarium*, *Rhizoctonia* and *Rhizopus* groups have been isolated from infected roots to cause seedling blight [5,6,7]. Among these fungus, the *Fusarium* genus was regarded as the major pathogens of rice seedling blight in China [8,9]. Thus, as a wide-ranging pathogens, *Fusarium oxysporum* was usually selected to induce seedling blight by many researchers [10,11].

Rice seedling blight is still treated using fungicides such as imazalil, tolclofos-methyl, fenaminosulf, liturium, and hymexazol, which are arguably effective against *Fusarium oxysporum* [12,13]. Due to growing environmental and health concerns, the need for eco-friendly alternatives to control this disease has become imperative [14,15].

In response to pathogens infection, plants can amass an array of formidable defense pathways using a variety of biological, chemical, or physical agents, which are known as resistance inducers or elicitors [16,17,18]. An earlier study showed that treating host plants with elicitors can increase secretion of defense-regulating enzymes such as phenylalanine ammonia lyase (PAL), peroxidase (POD), superoxide dismutase (SOD) and catalase (CAT) [19]. While PAL regulates host response to biotic and abiotic stress levels by increasing phenolic acids and phytoalexin production via the phenylpropanoid pathway, POD, SOD and CAT lower oxidative stress by acting as ROS scavengers [20]. Compared with other traditional disease control methods, the elicitors not only prevent plant injury but also reduce residue of chemicals in agricultural. It has features of low pollution, long duration and minimal side effects on crop quality. Therefore, it is an innovative approach to manage rice seedling blight [21,22].

Fluoro-substituted benzothiadiazole derivatives (FBT) is a novel elicitor similar to benzothiadiazole (BTH) structure, which was invented in China [23]. It can effectively induce high levels of defense mechanisms in many kinds of vegetable crops against soil-borne disease, as well as in the Chinese cabbage caused by *Plasmodiophora brassicae* [24]. Chitosan oligosaccharide (COS) derivatived from chitosan, has recently been shown to be an ideal delivery material of the elicitors regulating disease response and defense action [25,26]. In comparison to chitosan, COS is easily water-soluble and has good physio-chemical properties, qualifying it as a potential plant bio-vaccine. Thus, it is of prime interest to agricultural researchers [27]. In spite of these promising features, detailed molecular studies showing the use of FBT and COS in mitigating rice seedling blight are scarce.

The aim of this study was to evaluate the effect of FBT and COS treatments on *F. oxysporum* and elucidate the underlying molecular mechanisms by which they induce resistance against rice seedling blight using proteomics. It is anticipated that these findings will give further insights into the theory and application for FBT and COS as elicitors inducers for treating rice seedling blight.

## 2. Materials and Methods

### 2.1. Plant Growth Conditions

Rice (*Oryza sativa* L.) seeds (‘Qijing 2’), highly susceptible to blight, were obtained from the Rice research laboratory, Qiqihar branch of Heilongjiang Academy of Agricultural Sciences. Firstly, seed surfaces were disinfected with 70% ethanol for 1 min to achieve surface disinfection, subsequently incubated in 25 °C incubator until they sprouted to 5 mm, and finally washed with water and planted in 16 cm-diameter holes. All cultivations were cultivated in soil in the greenhouse of day 26 °C/night 20 °C with a 16 h light/8 h dark regime. The light intensity was 6000 LX.

### 2.2. Pathogen Inoculation

Standard pathogenic strain of *F. oxysporum* FO2016038 was provided by the Institute of Rice Research of the Northeast Agricultural University, Harbin, China. The pathogen was cultured on potato dextrose agar (PDA) medium at 4 °C. Conidial suspensions of the pathogen were prepared by flooding the 7-day-old culture dishes and incubated at 25 °C with sterile distilled water containing 0.1% Tween-20. The resulting zoospore concentration was adjusted to 1 × 10^6^ spores/mL with sterile distilled water using a hemocytometer to prepare the inoculum.

### 2.3. Elicitor Treatment and Sampling

Rice seedlings at 2-leaf stage were treated with 10 mL of one of three following elicitors by surface spraying: (1) sterile distilled water as the control (CK), (2) 50 mg/L FBT (1,2,3-benzothiadiazole-7-carboxylic acid-2,2,2-trifluoroethyl ester, purchased from Shanghai Taihe Chemical Co., Ltd., Shanghai, China) solution or (3) 100 mg/L COS (molecular mass: 1500–3000 Da, purchased from Hainan Zhengye Zhongnong High-tech Co., Ltd., Hainan, China) solution. After 2 days, they were then inoculated with 10 mL of conidial suspension (1 × 10^6^ spores/mL) by root-dip technique. Afterwards, tissue samples were immediately preserved in liquid nitrogen and stored at −80 °C until further analysis.

### 2.4. Efficiency of FBT and COS to Elicit Resistance against Seedling Blight

Disease incidence, disease index and root growth were determined at 1, 2, 3, 4, 5, 6 and 7 d after inoculation of conidial suspension. There are three replicates with 30 plants per replicate, and the experiment was conducted thrice. The disease investigation was performed as previously described by Wang et al. (Wang et al., 2009) (Table S1). The following formulas were used to determine the disease incidence and disease index, respectively:

$$\mathrm{Disease}\;\mathrm{incidence}\;\left(\%\right)\;=\;\frac{\mathrm{No}.\;\mathrm{of}\;\mathrm{diseased}\;\mathrm{plants}\;}{\mathrm{No}.\;\mathrm{of}\;\mathrm{total}\;\mathrm{investigated}\;\mathrm{plants}}\;\times \;100\%$$

$$\mathrm{Disease}\;\mathrm{index}\;=\;\frac{\Sigma \mathrm{No}.\;\mathrm{of}\;\mathrm{diseased}\;\mathrm{plants}\;\times \;\mathrm{grade}\;}{\mathrm{No}.\;\mathrm{of}\;\mathrm{total}\;\mathrm{investigated}\;\mathrm{plants}\;\times \;\mathrm{the}\;\mathrm{highest}\;\mathrm{grade}}\;\times \;100$$

Water on the surface of rice root was absorbed by filter paper, then it was dried at 105 °C for 15 min and baked to constant weight at 70 °C to determine the root dry weight. Root length, root surface area and root volume were measured with Epson root scanner and analyzed by WinRHIZO software.

### 2.5. Effect of FBT and OCT on Enzymatic Activity of Root

#### 2.5.1. Superoxide Dismutase (SOD) Assay

The activity of SOD was determined by measuring the inhibition of nitroblue tetrazolium (NBT) as earlier described by Beauchamp and Fridovich [28]. Briefly, fresh sample (0.5 g) was taken and 5 mL of 50 mM pre-cooled potassium phosphate buffer (pH 7.0) was added to extract the crude SOD enzyme solution. Afterwards a reaction mixture (9 mL) was prepared using the following −50 mM phosphate buffer (pH 7.8), 13 mM methionine, 75 μM NBT, 0.1 mM EDTA, 150 μL enzyme extract and 2 μM riboflavin which was added at the end. 3 mL of this mixture was poured in a tube, stirred and placed 30 cm below two 15 W fluorescent lamps. Reaction was induced by switching on the lamps for 15 min and stopped by switching off the lamps and placing a black cloth over the reaction tube. The control reaction mixture had no color. Reaction mixture absorbance was read at 560 nm. One unit of activity was defined as the amount of enzyme required to inhibit 50% of the NBT reduction rate in the controls containing no enzymes.

#### 2.5.2. Peroxidase (POD) Assay

POD activity was measured by following the method of Hammerschmidt et al. [29]. Fresh samples (0.5 g) were taken and 5 mL of 10 mM pre-cooled potassium phosphate buffer (pH 6.9) was added to extract the crude POD enzyme solution. Then, a reaction mixture consisted of 0.25% v/v guaiacol in 10 mM potassium phosphate buffer (pH 6.0) containing 100 mM hydrogen peroxide of which 3 mL was subsequently used. The crude enzyme (10 μL) was added to initiate the reaction and POD activity was evaluated using a spectrophotometer at a wavelength of 470 nm. The results were expressed on fresh weight basis as units (U) g^−1^. One unit of POD activity was defined as the amount of enzyme that causes an increase of 0.01 in the absorbance per minute at 470 nm.

#### 2.5.3. Catalase (CAT) Assay

The method of Bailly et al. [30] was used to determine CAT activity. Briefly, fresh samples (0.5 g) were taken and 5 mL of 100 mM pre-cooled potassium phosphate buffer (pH 7.0) was added to extract the crude CAT enzyme solution. The reaction mixture contained 3 mL of phosphate buffer along with 40 µL crude CAT extract was initiated by adding 40 µL of 10 mM H_2_O_2_. The activity of CAT was measured using a spectrophotometer (Hitachi U 2000, Tokyo, Japan) at 240 nm. CAT activity was expressed in terms of the change in absorbance at 240 nm in the linear phase of the slope (D240 min^−1^ g^−1^ fresh weight).

#### 2.5.4. Phenylalanine Ammonia-Lyase (PAL) Assay

The PAL activity was determined as earlier described by Beaudoin-Eagan and Thorpe [31]. Briefly, PAL enzyme was extracted with 5 mL of 25 mM pre-cooled Tris HCl buffer from 0.5 g fresh samples. Afterwards, 100 µL of the extracted enzyme was mixed with 900 µL of 50 mM L-Phenylalanine and 100 mM Tris HCl buffer solution (pH 8.01). Reaction was initiated by placing this mixture in a water bath at 40 °C for 2 h and stopped using 60 µL of 5 N HCl. The results were expressed on fresh weight basis as units (U) g^−1^. One unit of PAL acivity was defined as the amount of enzyme that causes an increase of 0.01 in the absorbance at 290 nm in 1 min.

### 2.6. Quantitative Analysis of Global Proteome

#### 2.6.1. Protein Sample Preparation

The detailed description of protein sample preparation including trypsin Digestion, tandem mass tags (TMT) labeling and HPLC fractionation can be found in the Supplementary Materials. 

#### 2.6.2. Liquid Chromatography Tandem Mass Spectrometry (LC−MS/MS) Analysis

Details of this procedure is available in the Supplementary Materials. Briefly, peptides were dissolved in 0.1% FA, directly loaded onto a reversed-phase pre-column (Acclaim PepMap 100, Thermo Scientific, Waltham, MA, China). Peptide separation was performed using a reversed-phase analytical column (Acclaim PepMap RSLC, Thermo Scientific). The peptides were subjected to NSI source followed by tandem mass spectrometry (MS/MS) in Q ExactiveTM plus (Thermo Scientific) coupled online to the UPLC. The mass spectrometry proteomics data have been deposited to the ProteomeXchange Consortium (http://proteomecentral.proteomexchange.org) via the iProX partner repository [32] with the dataset identifier PXD014979.

#### 2.6.3. Protein Identification and Screening of Differentially Expressed Proteins (DEPs)

The resulting MS/MS data were processed using Maxquant search engine software (v.1.5.2.8). Tandem mass spectra were searched against Phaffia_rhodozyma database concatenated with reverse decoy database. The Kyoto Encyclopedia of Genes and Genomes (KEGG) database was used to identify enriched pathways by a two-tailed Fisher’s exact test to test the enrichment of the DEPs against all identified proteins. Correction for multiple hypothesis testing was carried out using standard false discovery rate control methods. The pathway with a corrected *p*-value < 0.05 was considered significant. These pathways were classified into hierarchical categories according to the KEGG website. Cluster membership (Pearson algorithm) was constructed by a heat map using the “heatmap.2” function from the “gplots” R-package. 

### 2.7. Determination of Momilactone in Rice Root

Momilactone was measured by following the method of Kato-Noguchi et al. [33]. Briefly, rice root (5 g fresh weight) was homogenized with 50 mL of 80% (v/v) aqueous methanol and the homogenate was filtered through filter paper. The residue was homogenized again with 50 mL of methanol and filtered. The two filtrates were combined and evaporated in vacuo at 35 °C to give an aqueous residue. After evaporation, the methanol fraction was dissolved in 50% aqueous methanol (2 mL, v/v) and loaded onto a reverse-phase C18 Sep-Pak cartridge (Waters, Milford, MA, USA). The cartridge was first eluted with 50% aqueous methanol (15 mL) to remove impurities, and then with methanol (20 mL) to release momilactone. Momilactone was quantified by measuring its peak height on the chromatogram of High Performance Liquid Chromatography (HPLC) as described by Kato-Noguchi et al. [34].

### 2.8. Confirmation of the Infection-Responsive Expression Profiles by qRT-PCR

To validate the proteomic data results, qRT-PCR analysis was performed on 9 proteomic samples. Several genes that were co-expressed in both cultivars were analyzed by qRT-PCR at CK, FBT, and COS. The EASYspin Plus kit (Aidlab, Beijing, China) was used to extract the RNA of all samples following the manufacturer’s instructions, and 500 ng RNA was used for cDNA synthesis using the SuperScript III First-Strand Synthesis System for RT-PCR (Gene Denovo Biotechnology Co. Guangzhou, China) using Oligo(dT)20 primer. qRT-PCR reactions were run on the ABI PRISM^TM^ 7900HT Fast Real-Time PCR System (ABI) using SYRB^®^ GreenER^TM^ qPCR SuperMix (Invitrogen, Carlsbad, CA, USA). Reverse transcription was performed using 500 ng of RQ1 DNase (Promega, Madison, WI, USA)-treated total RNA, oligo dT and SuperScript III reverse transcriptase from the Invitrogen’s First-Strand cDNA Synthesis Kit according to the manufacturer’s instructions. The first-strand cDNA reaction was diluted 20 folds prior to qPCR and 5 μL of diluted cDNA was used as the PCR template. Reverse transcriptase negative controls were implemented for each PCR reaction to ensure that there is no genomic DNA contamination. The primer sequences used were shown in Table S2. Ct values were determined based on two biological replicates each with two technical replicates. Relative expression levels of target genes were calculated using the ΔΔCt method [35] and with the housekeeping gene EF1α mRNA as an internal standard. 

### 2.9. Statistical Analysis

All values obtained were expressed as mean ± standard deviation (SD). All experiments were performed at least thrice using independent assays. The statistical significance of data comparisons was determined using one-way analysis of variance (ANOVA), followed by Duncan’s multiple range test. Values of *p* < 0.05 were considered to be statistically significant.

## 3. Results

### 3.1. Efficacy of FBT and COS on Control of Seedling Blight

**Figure 1:**
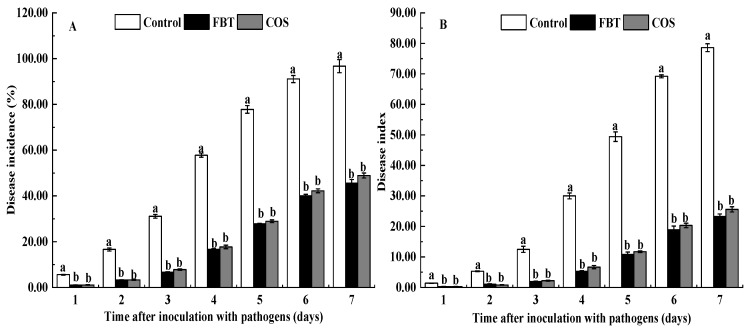
Effects of fluoro-substituted benzothiadiazole derivatives (FBT) and chitosan oligosaccharide (COS) on disease incidence and disease index caused by *F. oxysporum.* (**A**) disease incidence, and (**B**) disease index. The values were the means of three replicates of three different experiments. Values with different superscript letters were significantly different at *p* < 0.05.

As shown in Figure 1, the disease incidence and index in the rice roots of all groups were gradually increased following *F. oxysporum* inoculation (Figure 1). Compared with the control root, FBT and COS significantly (*p* < 0.05) reduced the disease incidence and development of disease symptoms caused by *F. oxysporum*.

### 3.2. Effect of FBT and COS on Growth Status of Root

**Figure 2:**
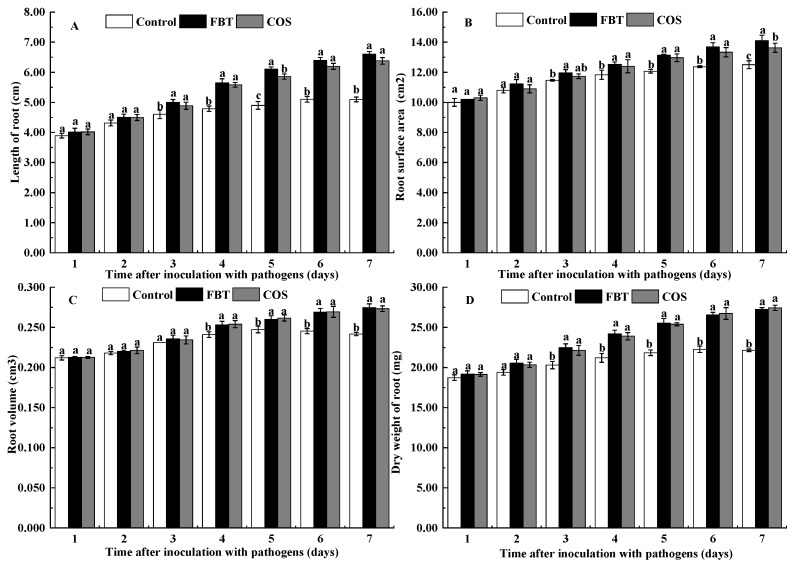
Effects of FBT and COS on growth of root caused by *F. oxysporum.* (**A**) root length, (**B**) root surface area, (**C**) root volume, and (**D**) root dry weight. The values were the means of three replicates of three different experiments. Values with different superscript letters were significantly different at *p* < 0.05.

As shown in Figure 2, there were no significant differences (*p* > 0.05) in root length, root surface area, root volume and root dry weight between two treatments with the control during the first 2 days after *F. oxysporum* incubation, respectively. However, after 4 d of incubation of pathogen, FBT and COS significantly (*p* < 0.05) increased the value of these parameters compared to the control root.

### 3.3. Effect of FBT and COS on Enzymatic Activities of Root

#### 3.3.1. Superoxide Dismutase (SOD) Assay

**Figure 3:**
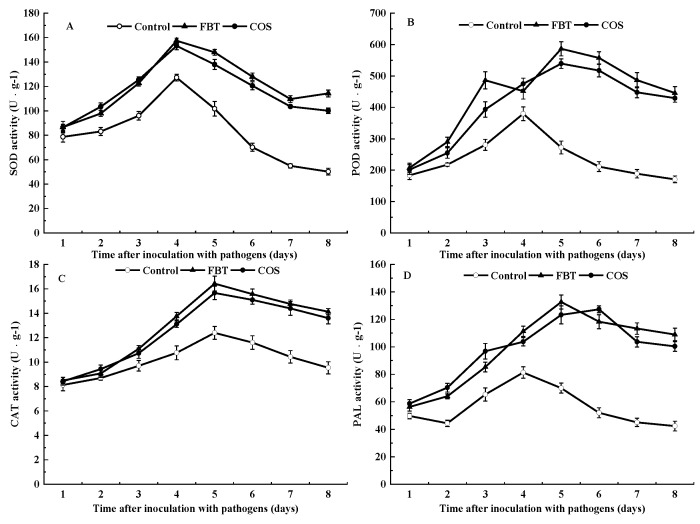
Effects of FBT and COS on activities of superoxide dismutase (SOD) (**A**), peroxidase (POD) (**B**), catalase (CAT) (**C**), and phenylalanine ammonia lyase (PAL) (**D**) in root. Data are expressed as the mean of triplicate assays. The values were the means of three replicates of three different experiments.

Root samples with and without elicitors had SOD activity. At all observed time intervals, SOD activity was significantly (*p* < 0.05) higher in FBT and COS-treated roots than that in control. In all root samples, the general trend was that SOD activity initially increased but subsequently decreased and peaked at day 4 of pathogen incubation (Figure 3A).

#### 3.3.2. Peroxidase (POD) Assay

Similar to the activity of SOD, the results of POD activity showed that all root groups with and without elicitors had POD activity, and it was significantly (*p* < 0.05) higher in FBT and COS-treated roots compared to control at all the observed time intervals. In FBT-treated roots, POD activity gradually increased and peaked at day 3, whereas, it appeared as a bimodal curve in COS-treated roots. Furthermore, POD activity in FBT-treated roots was higher than that in COS-treated roots at all tested time points except day 3 (Figure 3B), indicating that FBT had a better effect than COS throughout the study test time.

#### 3.3.3. Catalase (CAT) Assay

All roots with or without elicitors showed CAT activity. At all tested time points, CAT activity was significantly (*p* < 0.05) higher in FBT and COS-treated roots compared to the control root. The maximum CAT activity was found at 4 days in all the tested roots. Moreover, it was 1.33 and 1.26 fold higher in FBT and COS-treated roots than that in the control roots, respectively (Figure 3C). These findings were inconsistent with the results of peroxidase assay.

#### 3.3.4. Phenylalanine Ammonia-Lyase (PAL) Assay

Similar to previous assays, PAL activity occurred in all root categories with or without elicitors. Results showed that PAL activity was triggered by FBT and COS in root after inoculation of *F. oxysporum*, which was significantly (*p* < 0.05) higher than that in the control root (Figure 3D). These results indicated that the two elicitors could alleviate the rice seedling blight by regulating phenylpropanoid pathway.

### 3.4. Proteomic Characteristics of All Samples

**Figure 4:**
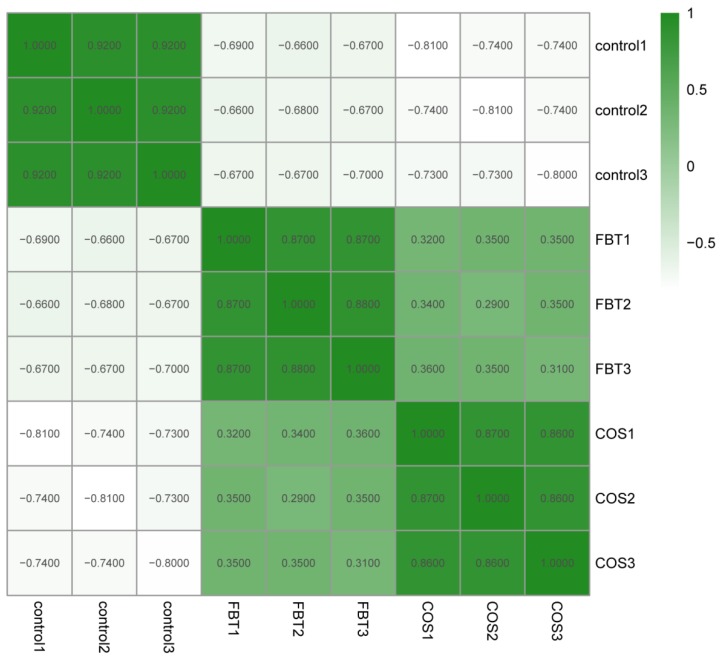
Heatmap showing Pearson correlation coefficients from all quantified proteins between each pair of samples.

To study variations in protein regulation in root samples induced by the two elicitors to resist *F. oxysporum*, TMT labling LC-MS/MS proteomic approach was used to measure the protein expression in rice roots after *F. oxysporum* inoculation. As shown in Figure 4, high correlations were observed among three replicates in the experimental and control groups with each other (R^2^ > 0.85), but correlation within treatment groups were lower. This suggested that protein expression profiles between biological replicates was consistent, and different protein expression profiles were likely in response to different kinds of elicitors.

### 3.5. Differential Expression and Biological Pathway Enrichment Analysis

**Figure 5:**
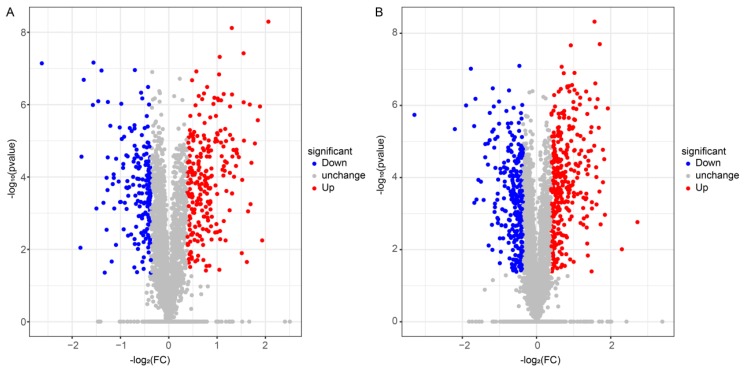
Volcano plots of Differentially Expressed Proteins (DEPs) in FBT-(**A**) and COS-(**B**) treated roots. DEPs were selected by adjusted *p*-value < 0.05. The x-axis shows the fold change in protein expression, and the y-axis shows the statistical significance of the differences. Grey dots indicate proteins without significantly differential expression; red dots denote significantly up-regulated proteins in FBT and COS-treated roots compared to the control root; and blue dots mean significantly down-regulated proteins in FBT and COS-treated roots compared to the control root.

Here, 922 and 1323 differentially expressed proteins (DEPs) were identified in FBT and COS-treated roots, respectively, compared with those in the control root after *F. oxysporum* inoculation. Moreover, 501 up-regulated proteins and 421 down-regulated proteins were identified between the FBT-treated root and the control root (Figure 5A), a total of 677 up-regulated genes and 646 down-regulated genes were found between the COS-treated root and the control root (Figure 5B).

**Figure 6:**
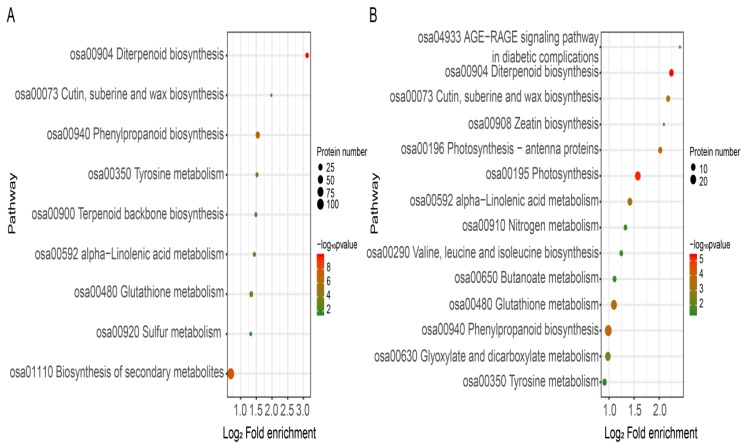
Bubble plot of KEGG pathway enrichment for DEPs in FBT-(**A**) and COS-(**B**) treated roots. The rich factor is calculated as the DEP number divided by the base number of any given pathway. Dot size denotes the number of proteins and dot color denotes the range of –log10 *p* value, and a lower –log10 *p* value indicates greater pathway enrichment.

The pathways that were enriched in the treated roots compared to the control roots are shown in Figure 6. FBT-treated roots had 150 DEPs out of 922 and these were assigned to 9 biological pathway annotations: ‘diterpenoid biosynthesis’, ‘biosynthesis of secondary metabolites’, ‘phenylpropanoid biosynthesis’, ‘glutathione metabolism’, ‘tyrosine metabolism’, ‘terpenoid backbone biosynthesis’, ‘alpha-linolenic acid metabolism’, ‘cutin, suberine and wax biosynthesis’ and ‘sulfur metabolism’ (Figure 6A). On the other hand, out of 1323 DEPs identified in COS-treated roots, 187 were assigned to 14 enrichment pathways: ‘diterpenoid biosynthesis’, ‘photosynthesis’, ‘phenylpropanoid biosynthesis’, ‘photosynthesis-antenna proteins’, ‘cutin, suberine and wax biosynthesis’, ‘glutathione metabolism’, ‘alpha-Linolenic acid metabolism’, ‘glyoxylate and dicarboxylate metabolism’, ‘AGE-RAGE signaling pathway in diabetic complications’, ‘zeatin biosynthesis’, nitrogen metabolism’, ‘valine, leucine and isoleucine’, and ‘tyrosine metabolism’ (Figure 6B). Interestingly, the ‘diterpenoid biosynthesis’ pathway, having the smallest Q value, was shown to be significantly enriched in both FBT and COS treatments compared to the other pathways. Furthermore, ‘diterpenoid biosynthesis’, ‘alpha-Linolenic acid metabolism’, ‘phenylpropanoid biosynthesis’, ‘cutin, suberine and wax biosynthesis’ and ‘tyrosine metabolism’ were shared by the two groups, implying that FBT and COS may have the same molecular mechanisms in the induction of resistance in rice seedling blight.

### 3.6. Specific Pathway of Diterpenoid Biosynthesis Analysis

**Figure 7:**
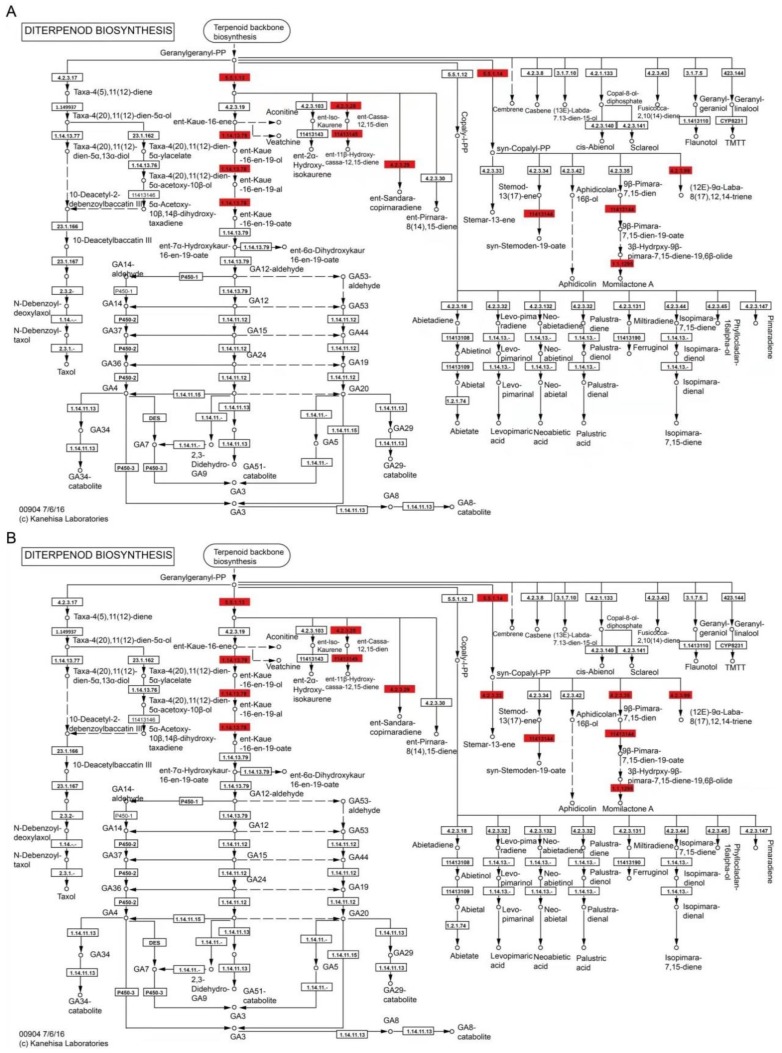
Illustration of KEGG pathway of diterpenoid biosynthesis in COS-(**A**) and FBT-(**B**) treated roots. Red signifies up-regulated proteins in FBT and oligochitosan-treated roots compared to control root.

Compared to the control root, the diterpenoid biosynthesis pathway in the COS-treated roots contained eight DEPs (Figure 7). These include two ent-copalyl diphosphate synthases [EC:5.5.1.13] (Os02t0571100-01 and Os02t0570900-00), an ent-cassa-12,15-diene synthase [EC:4.2.3.28] (Os02t0570400-01), a ent-kaurene oxidase [EC:1.14.14.86] (Os06t0569500-01), two ent-cassa-12,15-diene 11-hydroxylases [EC:1.14.14.112] (Os02t0569900-01 and Os02t0569400-01), a sandaracopimaradiene/labdatriene synthase [EC:4.2.3.29 4.2.3.99] (Os12t0491800-01), a syn-copalyl-diphosphate synthase [EC:5.5.1.14] (Os04t0178300-02), a 9-beta-pimara-7,15-diene oxidase [EC:1.14.14.111] (Os04t0178400-01), and a momilactone-A synthase [EC:1.1.1.295] (Os04t0179200-01). In addition, a stemar-13-ene synthase [EC:4.2.3.33] (Os11t0474800-01) and a syn-pimara-7,15-diene synthase [EC:4.2.3.35] (Os04t0179700-01) were found in the FBT-treated treated roots compared to the control root (Figure 7B). These results show that all DEPs were upregulated in both treatments.

**Figure 8:**
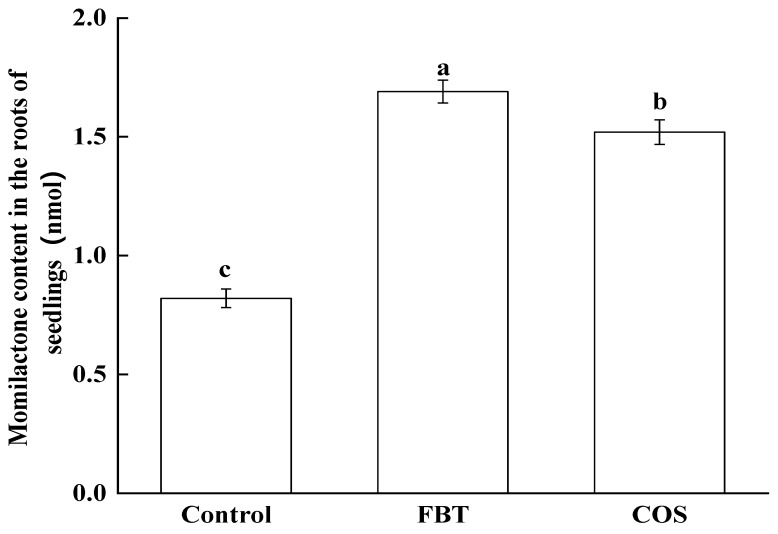
Concentrations of momilactone in rice root. The values were the means of three replicates of three independent experiments. Values with different superscript letters were significantly different at *p* < 0.05.

In order to confirm the prediction made by the proteomic changes, the level of momilactone in rice root in all groups was measured by HPLC. As shown in Figure 8, compared with the control group, FBT and COS treatment significantly (*p* < 0.05) increased the concentrations of momilactone in the roots. Furthermore, it in the FBT-treated roots was higher than that in the COS-treated roots. This result was consistent with the profile of proteomics. 

### 3.7. Confirm Unigenes Expression Using Real-Time Quantitative Reverse Transcription PCR

**Figure 9:**
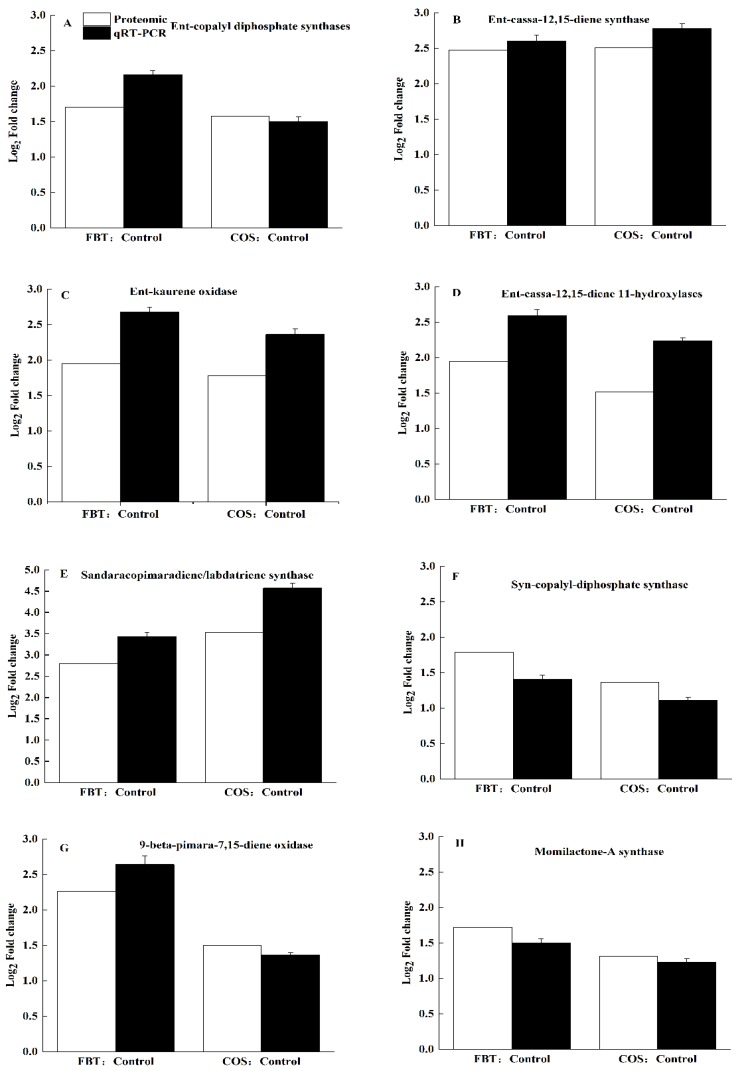
Verification of the proteomic results by qRT-PCR. White bar: proteomic data for the proteins. Black bar: qRT-PCR results for the proteins. The values were the means of three replicates of three different experiments. (**A**) ent-copalyl diphosphate synthases, (**B**) ent-cassa-12,15-diene synthase, (**C**) ent-kaurene oxidase, (**D**) ent-cassa-12,15-diene 11-hydroxylases, (**E**) sandaracopimaradiene/labdatriene synthase, (**F**) syn-copalyl-diphosphate synthase, (**G**) 9-beta-pimara-7,15-diene oxidase, and (**H**) momilactone-A synthase.

Quantitative real-time PCR (qRT-PCR) was performed to validate our earlier obtained proteomics results. Here, eight DEPs, which are in the diterpenoid biosynthesis pathway-ent-copalyl diphosphate synthases, ent-cassa-12,15-diene synthase, ent-kaurene oxidase, ent-cassa-12,15-diene 11-hydroxylases, sandaracopimaradiene/labdatriene synthase, syn-copalyl-diphosphate synthase, 9-beta-pimara-7,15-diene oxidase and momilactone-A synthase. After confirming our results, all selected 8 DEPs exhibited similar expression patterns as observed in proteomic data, demonstrating that proteomic results were accurate in this study (Figure 9).

## 4. Discussion

In response to numerous pathogen attacks, plants amass a formidable defense system by using a number of biotic and abiotic resources that serve as elicitors, a defense strategy largely known as induced resistance [36,37]. This type of defense strategy has garnered tremendous research interests because it is not harmful to humans and poses no threat to the environment. It is a safe approach in combating a host of plant diseases [38,39]. Therefore, the present study aims to elucidate the resistance-eliciting efficiency of the FBT and COS treatments against rice seedling blight from biochemical and molecular perspectives.

In this study, FBT and COS treatments significantly reduced both disease incidence and index (Figure 1). This suggests that FBT and COS could be potential elicitors that increase rice resistance to *F. oxysporum* attack. FBT and COS treatments also prevented root inhibition caused by this fungus. (Figure 2). In addition, there was a corresponding increase in the activities of defense enzymes, such as POD, SOD, CAT and PAL (Figure 3). Among these enzymes, POD, SOD and CAT are important components of antioxidant systems to develop a broad range of defense responses to cope with pathogenic infections [40]. POD plays an important role in generating H_2_O_2_ as part of the defense response and confers resistance to a wide range of plant pathogens [41]. A previous study also found that POD is implicated in the polymerization of monolignols into lignin and cell wall reinforcements after pathogen attack [42]. SOD can transform superoxide radical anions (O^2−^) to H_2_O_2_ [28], CAT can turn H_2_O_2_ into water in plant cell [43]. PAL plays a critical role in the phenylpropanoid pathway and in the response and regulation of biotic and abiotic stresses [44,45]. Our results showed that FBT and COS could increase the activities of POD, SOD, CAT and PAL (Figure 3), these results were in line with previous investigations. Similarly, it was reported that at all tested time points, the activities of POD, SOD and CAT was significantly higher in chitosan nano-treated seedlings compared to the pathogen-inoculated seedlings in pearl millet cultivars [26]. In a related study, chitosan acted as an elicitor to increase CAT activity, giving improved systemic resistance of potato tuber [46]. The phenylpropanoid biosynthesis pathway was also recently implicated in resistance conferment among citrus fruit samples in response to three elicitors—Salicylic acid, *Pichia membranaefaciens* and oligochitosan [25]. The above fingdings established the basis that FBT and COS could be used as elicitors to promote increases in the potential immunity of rice to ameliorate seedling blight.

In recent years, proteomic techniques have been increasingly useful in gene screening protocols, functional genes discovery and understanding complex molecular mechanisms in the ever-evolving plant-pathogen interaction [47]. The current study used the TMT-based proteomic technique in order to mine some of the underlying molecular mechanisms by which FBT and COS induce resistance against rice seedling blight. The results of correlation coefficients showed that a good consistency of protein expression profiles between biological replicates, suggesting that our experiment designs and proteomic data were reliable (Figure 4). A total of 922 and 132 DEPs were found in FBT and COS-treated roots, respectively, compared with those in the control roots after *F. oxysporum* inoculation, indicating that dfifferent kinds of elicitors are likely in response to different protein expression profiles (Figure 5). In addition, the KEGG database (two-tailed Fisher’s exact test) was used to assess DEP functions. Our results showed that 14 and 9 enriched pathways were observed in FBT and COS-treated roots after *F. oxysporum* inoculation (Figure 6).

Based on Q value, the diterpenoid biosynthesis pathway was thus selected for further study and results show that all DEPs enriched into this pathway were up-regulated in FBT and COS-treated roots (Figure 7), resulting in the increased secretion of momilactone (Figure 8). As an allelochemical in rice and moss plants with a 19, 6 β-lactone structure [48,49], *Momilactone* (A and B) has been proposed as environmentally-safe fungicidal and bacteriostatic agents [50]. Previous studies on its antifungal properties against the highly destructive rice blast pathogen *Piricularia oryzae* [49,51], as well as an integral part of the defense system of the moss *Hypnum plumaeforme* [52,53,54,55], strongly support the findings of the present study. In addition, momilactones A and B significantly suppressed *F. oxysporum* and *F. solani* activities in vitro [56]. Although the biological importance of momilactones and its defense role in rice and mosses have been abundantly reported in the past, it was not until recently that the molecular basis for some of these functionalities were unraveled [57,58,59,60,61]. This resulted in *Momilactose* (A and B) being officially patented as non-chemical herbicides a little over a decade ago, giving more credence to their value in green agriculture research [62]. The present study demonstrates that the activation of the diterpenoid biosynthesis pathway plays an important role in resistance induction against rice seedling blight using FBT and COS treatments. More so, the terpenoid biosynthesis pathway can regulate the synthesis of precusor substances of momilactone. While analyses of FBT-treated roots showed DEPs upregulation in the terpenoid biosynthesis pathway (Figure S1), no upregulated proteins were found in COS-treated root. This may be the reason why there were higher resistance levels in the former compared to the latter. Finally, some POD activities were up-regulated while others were down-regulated after *F. oxysporum* inoculation (Table S3). Both treatments elevated PAL activities, but the expression level of PAL was not altered. The basis for this divergence is unclear at this time and thus further studies are recommended.

In summary, FBT and COS significantly reduced both the disease incidence and index, improved the growth status of root, and enhanced defense enzyme activities induced by *F. oxysporum*. Both elicitors significantly induced the changes of proteins involved in diterpenoid biosynthesis pathway, resulting in the increase of related enzyme activities and the accumulation of the antimicrobial substance, momilactone. It can be concluded that FBT and COS could be eco-friendly elicitors for resistance against rice seedling blight, which could significantly boost rice productivity to meet the nutritional needs of a rising global population in China.

## Supplementary Materials

The following are available online at https://www.mdpi.com/2223-7747/8/12/538/s1, Figure S1: Illustration of KEGG pathway of terpenoid backbone biosynthesis in oligochitosan-treated roots. Red means up-regulated proteins in FBT and COS-treated roots compared to control root, Table S1: Grading standard of rice seedlings blight, Table S2: Primer sequences of Protein for RT-qPCR, Table S3: DEPs annotated as peroxidase in FBT and COS-treated.
