# Supplementary material for: Preventive Effects of Fluoro-Substituted Benzothiadiazole Derivatives and Chitosan Oligosaccharide against the Rice Seedling Blight Induced by Fusarium oxysporum
Source: Plants (Basel). 2019 Nov 24;8(12):538. doi: 10.3390/plants8120538 (PMC6963531; doi:10.3390/plants8120538)
Supplement: Supplementary file 1 [file plants-08-00538-s001.pdf]

Preventive Effects of Fluoro-substituted Benzothiadiazole Derivatives and Chitosan Oligosaccharide against the Rice Seedling Blight Induced by *Fusarium oxysporum*

The Detailed Description of Sample Preparation for Proteomics

1.1 Trypsin Digestion

For digestion, the protein solution was reduced with 10 mM DTT for 1 h at 37 °C and alkylated with 20 mM IAA for 45 min at room temperature in darkness. For trypsin digestion, the protein sample was diluted by adding 100 mM TEAB to urea concentration less than 2M. Finally, trypsin was added at 1:50 trypsin-to-protein mass ratio for the first digestion overnight and 1:100 trypsin-to-protein mass ratio for a second 4 h-digestion. Approximately 100 μg protein for each sample was digested with trypsin for the following experiments.

1.2 TMT Labeling

After trypsin digestion, peptide was desalted by Strata X C18 SPE column (Phenomenex) and vacuum-dried. Peptide was reconstituted in 0.5 M TEAB and processed according to the manufacturer's protocol for 6-plex TMT kit. Briefly, one unit of TMT reagent (defined as the amount of reagent required to label 100 μg of protein) were thawed and reconstituted in 24 μl ACN. The peptide mixtures were then incubated for 2 h at room temperature and pooled, desalted and dried by vacuum centrifugation.

Table 1 Labeling information

Sample Groups	Labeling information
CK	126
OCN	127
FBT	128

1.3 HPLC Fractionation

The sample was then fractionated into fractions by high pH reverse-phase HPLC using Agilent 300 Extend C18 column (5 μm particles, 4.6 mm ID, 250 mm length). Briefly, peptides were first separated with a gradient of 2% to 60% acetonitrile in 10 mM ammonium bicarbonate pH 10 over 80 min into 80 fractions. Then, the peptides were combined into 18 fractions and dried by vacuum centrifuging.

1.4 LC-MS/MS Analysis

Peptides were dissolved in 0.1% FA, directly loaded onto a reversed-phase pre-column (Acclaim PepMap 100, Thermo Scientific). Peptide separation was performed using a reversed-phase analytical column (Acclaim PepMap RSLC, Thermo Scientific). The gradient was comprised of an increase from 6% to 22% solvent B (0.1% FA in 98%

ACN) over 22 min, 22% to 36% in 10 min and climbing to 85% in 5 min then holding at 85% for the last 3 min, all at a constant flow rate of 400 nl/min on an EASY-nLC 1000 UPLC system, The resulting peptides were analyzed by Q ExactiveTM plus hybrid quadrupole-Orbitrap mass spectrometer (ThermoFisher Scientific).

The peptides were subjected to NSI source followed by tandem mass spectrometry (MS/MS) in Q ExactiveTM plus (Thermo) coupled online to the UPLC. Intact peptides were detected in the Orbitrap at a resolution of 70,000. Peptides were selected for MS/MS using NCE setting as 30; ion fragments were detected in the Orbitrap at a resolution of 17,500. A data-dependent procedure that alternated between one MS scan followed by 20 MS/MS scans was applied for the top 20 precursor ions above a threshold ion count of 1E4 in the MS survey scan with 30.0s dynamic exclusion. The electrospray voltage applied was 2.0 kV. Automatic gain control (AGC) was used to prevent overfilling of the orbitrap; 5E4 ions were accumulated for generation of MS/MS spectra. For MS scans, the m/z scan range was 350 to 1800. Fixed first mass was set as 100 m/z.

Table S1 Grading standard of rice seedlings blight

Grade	Incidence
0	No symptoms
1	Disease spots at stem base are small and occupy below1/4 of stem circumference
2	Diseased spots at stem base are larger and occupy 1/4-1/2 of stem circumference
3	Diseased spots at stem base are larger and occupy 1/2-3/4 of stem circumference
4	Diseased spots at the base of stem occupied all stem circumference and plant die

Table S2 Primer sequences of Protein for RT-qPCR.

KEGG description	Protein accession	Genes	Primer sequence (5'-3')
1.ent-copalyl diphosphate synthases	Os02t0570900-00		F: TATGATGACCCTGCCTTGCA<br>R: CTATGGAGCAAGGTGGTCGG
2.ent-cassa-12,15-diene synthase	Os02t0570400-01		F: CAGGCCGTGAAGAGATCGAG<br>R: AGCCCTTAGCGTCGAAAAGC
3.ent-kaurene oxidase	Os06t0569500-01		F: CGTGCCACATGGACGAGAAG<br>R: CCGAACGCCATCGTCTTGTA
4.ent-cassa-12,15-diene 11-hydroxylases	Os02t0569900-01		F: CGTTCCACCACAAGCTCAGG<br>R: CTTGGTGTACGCCTCGATGG
5.sandaracopimaradiene/labdatriene synthase	Os12t0491800-01		F: TGCGTGCTCTCACTGACAGC<br>R: AACTCCGCATCTCCACCATG
6.syn-copalyl-diphosphate synthase	Os04t0178300-02		F: GCCGGTCTTCACTGCATCAT<br>R:GAAGGTGTAGGAACGGCCGT
7.9beta-pimara-7,15-diene oxidase	Os04t0178400-01		F: TCAAGGAGACCCTGAGGCTG<br>R:TCTGGTGCCCTCCACAATCT
8.momilactone-A synthase	Os04t0179200-01		F: GAACTCGGCGAACCTGAAGG<br>R:AAGCTGCTGTTGACGACGGA

Table S3 DEPs annotated as peroxidase in FBT and COS-treated

Protein accession	Protein description	FBT/CK Ratio	OCN/CK Ratio
Os01t0205900-02	Similar to Class III peroxidase GvPx2b (Fragment).	1.019	1.055
Os01t0263300-01	Similar to Peroxidase 72 precursor (EC 1.11.1.7) (Atperox P72) (PRXR8) (ATP6a).	0.984	0.884
Os01t0270300-02	Similar to Cationic peroxidase isozyme 40K precursor.	0.982	0.932
Os01t0294500-00	Similar to Class III peroxidase 9.	1.359	0.967
Os01t0294700-01	Haem peroxidase, plant/fungal/bacterial family protein.	0.832	0.818
Os01t0326000-01	Similar to Peroxidase (Fragment).	1.019	0.913
Os01t0326300-01	Haem peroxidase, plant/fungal/bacterial family protein.	1.323	1.105
Os01t0327100-01	Haem peroxidase family protein.	1.361	1.400
Os01t0327400-01	Similar to Peroxidase (Fragment).	1.343	1.271
Os01t0378100-01	Plant peroxidase domain containing protein.	1.050	1.023
Os01t0543100-01	Similar to Peroxidase 72 precursor (EC 1.11.1.7) (Atperox P72) (PRXR8) (ATP6a).	1.019	0.930
Os01t0962700-01	Similar to Peroxidase 12 precursor (EC 1.11.1.7) (Atperox P12) (PRXR6) (ATP4a).	1.530	1.420
Os02t0192700-02	Similar to Thioredoxin peroxidase.	0.872	1.162
Os02t0236600-01	Peroxidase P7 (EC 1.11.1.7) (TP7).	0.727	0.730
Os02t0236800-02	Similar to Peroxidase (EC 1.11.1.7).	0.846	0.875
Os02t0240100-01	Similar to Peroxidase 2 (Fragment).	1.451	1.115
Os02t0240300-01	Similar to Class III peroxidase GvPx2b (Fragment).	1.157	0.974
Os03t0121200-03	Similar to Class III peroxidase 33.	1.330	1.090
Os03t0121300-01	Similar to Peroxidase 1.	1.602	1.467
Os03t0235000-01	Similar to Peroxidase.	1.560	1.564
Os03t0339400-01	Haem peroxidase, plant/fungal/bacterial family protein.	0.937	0.634
Os03t0368300-01	Similar to Peroxidase 1.	1.297	1.018
Os03t0368900-01	Haem peroxidase family protein.	0.655	0.555
Os03t0762300-02	Similar to peroxidase 51.	0.711	0.660
Os04t0423800-01	Peroxidase (EC 1.11.1.7).	0.648	0.642
Os04t0465100-01	Haem peroxidase, plant/fungal/bacterial family protein.	0.983	0.908

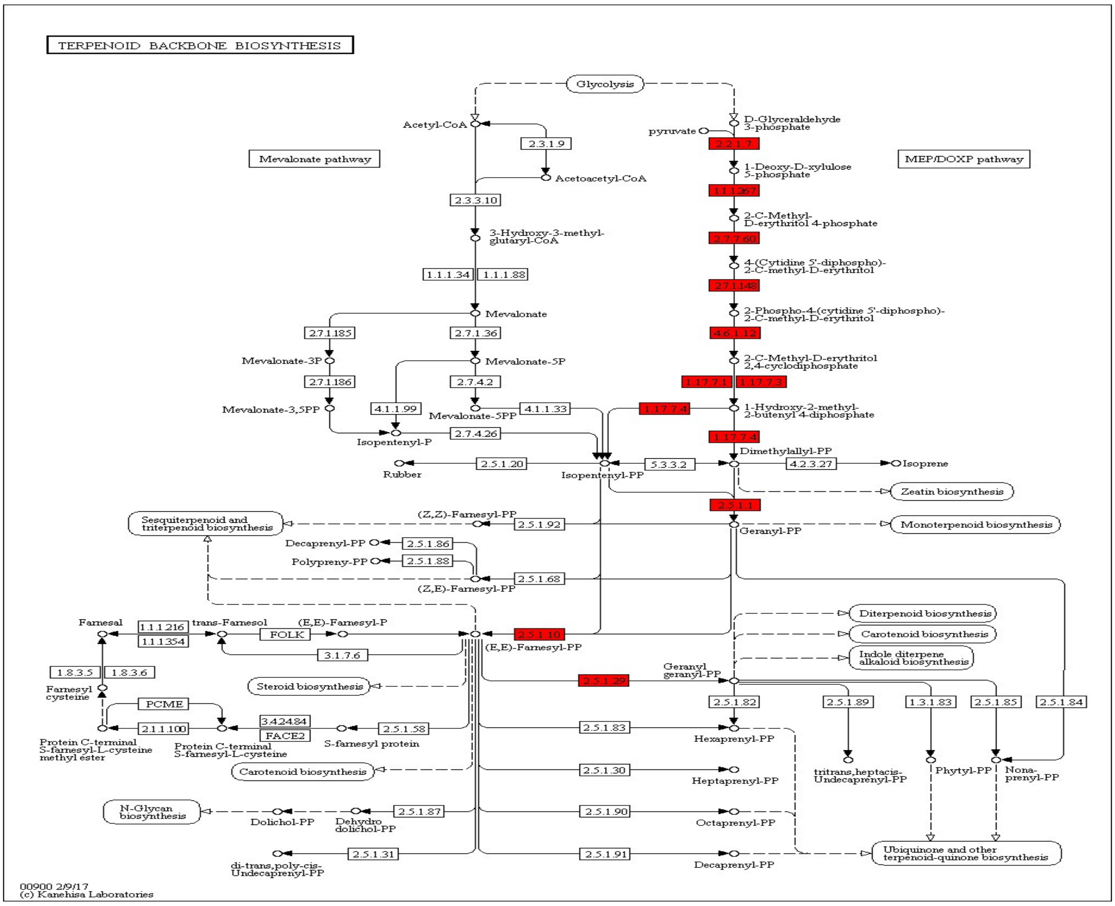

Figure S1 Illustration of KEGG pathway of terpenoid backbone biosynthesis in oligochitosan-treated roots. Red means up-regulated proteins in FBT and COS-treated roots compared to control root.
